# Fasting Therapy Contributes to the Improvement of Endothelial Function and Decline in Vascular Injury-Related Markers in Overweight and Obese Individuals via Activating Autophagy of Endothelial Progenitor Cells

**DOI:** 10.1155/2020/3576030

**Published:** 2020-07-27

**Authors:** Jiapan Sun, Tingying Zhang, Li Zhang, Bin Ke, Jian Qin

**Affiliations:** ^1^Department of Traditional Chinese Medicine, The First Affiliated Hospital, Sun Yat-Sen University, Guangzhou, Guangdong 510080, China; ^2^Department of Traditional Chinese Medicine, The Seventh Affiliated Hospital, Sun Yat-Sen University, Shenzhen, Guangdong 518107, China; ^3^Department of VIP Ward, Sun Yat-sen University Cancer Center, Guangzhou, Guangdong 510060, China

## Abstract

**Background:**

High body mass index- (BMI-) related vascular injury contributes to the pathogenesis of the atherosclerotic cardiovascular disease (ASCVD). Rigorous calorie restriction is one of the major lifestyle interventions to reduce vascular risk in overweight or obese individuals. However, the effects of fasting therapy (FT) on vascular function and the mechanism are still unclear. This study was aimed to investigate the impacts of FT on endothelial function, arterial stiffness, and circulating arterial damage parameters in overweight and obese individuals and possible mechanism.

**Methods:**

Overweight and obese individuals participated in FT intervention (7-day very low calorie diet). Arterial function including brachial arterial flow-mediated dilation (FMD), brachial-ankle pulse wave velocity (baPWV), vascular injury-related markers including trimethylamine N-oxide (TMAO), and leptin and endothelial microparticles (EMPs) were assessed. Endothelial progenitor cells (EPCs) of these participants were isolated and cultured to investigate EPCs function. mRFP-GFP-LC3 confocal microscopy scanning and western blot were carried out to determine autophagy.

**Results:**

After FT, body weight and BMI significantly decreased (81.76 ± 12.04 vs. 77.51 ± 12.06 kg, *P* < 0.01; 29.93 ± 2.82 vs. 28.47 ± 2.83 kg/m^2^, *P* < 0.01). FT remarkably improved FMD (5.26 ± 1.34 vs. 6.25 ± 1.60%, *P*=0.01) while baPWV kept unchanged. TMAO and leptin levels decreased (3.96 ± 1.85 vs. 2.73 ± 1.33 *μ*mol/L, *P*=0.044; 6814 ± 2639 vs. 3563 ± 2668 *μ*mol/L, *P* < 0.01). EMPs showed a decreased tendency. EPCs function was significantly improved, autophagy fluorescence intensity was enhanced, and the level of Beclin1, Atg5, LC3 II/I also increased after starvation in vitro, and the effects were blocked by autophagy inhibitor.

**Conclusions:**

Our present study demonstrated for the first time that FT markedly improves endothelial function and reduces the levels of arterial injury markers through improving EPCs function via activating autophagy. These findings provide a novel insight into FT as a lifestyle intervention strategy to promote the maintenance of vascular homeostasis in overweight or obese individuals. The trial was registered with ChiCTR1900024290.

## 1. Introduction

The prevalence of overweight and obesity is rising rapidly worldwide. High body mass index (BMI) is among the leading causes of elevated morbidity and mortality for atherosclerotic cardiovascular disease (ASCVD). Globally, it is estimated that 4 million deaths are caused by high BMI, more than two-thirds of which are related to ASCVD [1]. Impaired vascular function is the initial stage of ASCVD onset and predicts future adverse cardiovascular events [2]. Numerous reports support that overweight and obesity result in abnormal vascular function. Therefore, the maintenance of vascular function becomes an important target to reduce ASCVD in overweight and obese individuals [3–7].

Accumulating evidence indicates that the abnormal vascular function in overweight and obesity are mainly manifested as impaired endothelial function [4–6] and enhanced arterial stiffness [7] which can be evaluated by brachial arterial flow-mediated dilation (FMD) and brachial-ankle pulse wave velocity (baPWV). Moreover, circulating molecules such as trimethylamine N-oxide (TMAO), endothelial microparticles (EMPs), and leptin, have been identified as biomarkers of vascular injury in obesity. TMAO is derived from gut microbiome metabolism of choline to trimethylamine, and EMPs are kind of vesicular structures shed from activated or apoptotic endothelial cells. Besides, leptin is a circulating hormone secreted by adipocytes that regulate food-intake and glycolipid metabolism. All these three acknowledged biomarkers are abnormally increased in obesity [8–15]. Hence, developing a therapeutic approach to synthetically improve endothelial function, arterial stiffness, and vascular injury-related markers might have important clinical implications in restoring vascular homeostasis of overweight and obese individuals.

Rigorous calorie restriction is an important lifestyle to reduce vascular risk and may help prevent the incidence of ASCVD in high BMI persons [16, 17]. Fasting treatment which can be carried out by drinking water only or having very low calorie diet has been proved to be an effective regimen to lose weight and restore metabolic disorders [18]. Our previous study demonstrated beneficial effects of fasting therapy (FT) on weight loss, insulin sensitivity, blood pressure, and lipid profiles in obese patients with metabolic disorders [19, 20]. However, to date, limited evidence is available to address the effect of FT on vascular function in overweight and obese individuals and possible mechanism.

Endothelial progenitor cells (EPCs), initially defined as circulating bone marrow-derived CD34-positive/KDR (kinase domain receptor)-positive cells [21], protect the physiological structure and function of the endothelium and maintain vascular homeostasis [22]. However, studies show that the depletion and dysfunction of the circulating EPCs are thought to be the foundation of vascular dysfunction in obesity [23–25]. Therefore, the upregulation of the functional potential of EPCs is a pivotal target to repair vascular function in patients with metabolic disorders.

Autophagy is a highly conserved physiological process controlling endothelial homeostasis in vascular beds [26]. Studies showed that autophagy defect results in endothelial dysfunction of patients with obesity and metabolic disorders, promoting the progression of ASCVD [26–28]. Fasting is a classical and acknowledged method to regulate autophagy [29–31], which may probably make contributions to EPCs function.

Based on the data mentioned above, we hypothesized that the FT would improve arterial function in overweight and obese individuals and reduce circulating vascular injury-related biomarkers via regulating autophagy to repair EPCs function. To address these assumptions, overweight and obese individuals who were enrolled in this pilot study received FT. FMD and baPWV as well as circulating arterial damage parameters such as TMAO, EMPs, and leptin, were evaluated. EPCs of these participants were isolated and cultured to investigate EPCs function and autophagy level after starvation. The present study may provide novel insight into the beneficial effects of FT as a lifestyle intervention strategy to maintain vascular homeostasis and retard the pathogenesis of high BMI-induced ASCVD.

## 2. Subjects and Methods

### 2.1. Subjects

Thirteen overweight or obese individuals (BMI≥25 kg/m^2^) aged between 25 and 65 were recruited to participate in this study from The First Affiliated Hospital, Sun Yat-Sen University. Exclusion criteria were persons with abnormal heart, liver, or kidney function, secondary hypertension, essential hypertension above the second grade, insulin use, severe gastric bleeding, cancers, blood diseases, active tuberculosis, and women during pregnancy, lactation or menstruation. All subjects were given written informed consent prior to study participation. Ethics approval was granted by the ethical committee of The First Affiliated Hospital, Sun Yat-Sen University in Guangzhou ([2019] 085). The trial was registered in Chinese Clinical Trial Registry of WHO (ChiCTR1900024290).

### 2.2. FT Intervention

**Figure 1:**
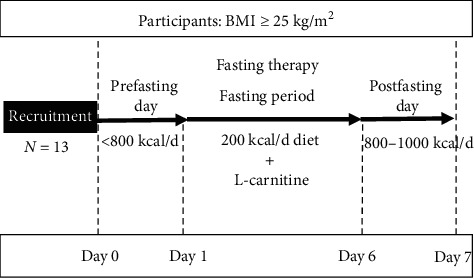
FT protocol. FT lasted for 7 days consisting of 3 phases (prefasting day, fasting period, and postfasting day). Measurements were taken at the time points of the 1^st^ and 7^th^ day of FT. FT: fasting therapy.

The open-label, small sample size, self-controlled trial was conducted at The First Affiliated Hospital, Sun Yat-Sen University. Participants received the FT, blood samples were collected, and arterial function examination was taken at the first and the last day of FT (see Figure 1).

FT lasted for 7 days including 1 day with moderate calorie restriction (prefasting day, <800 kcal/d, fruits and vegetables), 5 days of intense calorie restriction (fasting period, 200 kcal/d), and 1 day with the stepwise reintroduction of a normal diet (postfasting day, 800–1000 kcal/d, semiliquid or semisolid food). Each subject was given 10–20 g of thenardite powder on the first day of the fasting period for bowel-cleansing. During the fasting period, patients were asked to drink at least 2 L of mineral water, have 200 kcal/d diet each day consisting of one serving of the liquid meal replacement and two cups of light vegetable soup, and receive L-carnitine (Lanling Pharmaceutical CO., LTD, Changzhou, China) 2 g, bid (9 a.m. and 4 p.m), i.v. infusion (dissolving 2 g L-carnitine into 20 mL saline). Each serving of the liquid diet provided approximately 150 kcal: 7.9 g protein, 2.8 g fat, and 22 g carbohydrate. The liquid diet was prepared by mixing the powdered product with 250 ml water and was to be consumed at dinner. Two bowls of vegetable soup provided about 50 kcal and were given at breakfast and lunch. All patients were required to engage in low-level physical activity consisting of 2 h of slow walking per day. Furthermore, participants were recommended to avoid alcohol, coffee, or tea during the study. All participants were compulsorily hospitalized during FT intervention [19, 20].

### 2.3. FMD Measurement

Each subject underwent vascular function examination by trained sonographers, performed between 8 and 11 a.m.; they were recommended to rest for 10 min or longer and were examined in a quiet, temperature-controlled room. FMD was examined through noninvasive ultrasound scan (UNEXEF38G, Sakae, Japan); it could dynamically record artery diameter, at rest (baseline) and reactive hyperemia period produced by using the cuff inflated to the greater of 50 mmHg above systolic pressure or 200 mmHg for 5 min as previously described [32]. FMD was calculated as follows:

(1) $$\begin{array}{c} \text{FMD}\left(\%\right)=\frac{\left(\text{maximum diameter}-\text{diameter at rest}\right)}{\left(\text{diameter at rest}\right)}*100. \end{array}$$

### 2.4. baPWV Measurement

Arterial stiffness was assessed with baPWV in supine individuals after rest. baPWV was measured by using an automatic device (VP-2000, Colin, Japan) as reported previously [33] and was expressed as centimeters per second (cm/sec). The measurement of baPWV is generally accepted as the simplest, robust, and reproducible method to represent arterial stiffness [34].

### 2.5. Biochemical Measures

Blood samples were collected between 08:00 and 10:00 in the morning after fasting overnight. TMAO was tested by using circulating plasma. Firstly, 10 *μ*L of internal standard solution (TMAO-d9, 5 *μ*g/mL) was added to 100ul plasma; then 300 *μ*l of acetonitrile for protein precipitation was added, vortexed for 1 min, centrifugated at 1000 rpm for 5 minutes, 4°C; at last, we took 200 *μ*L of the supernatant in the vial for HPLC-MS/MS analysis [35]. Leptin was tested by using circulating serum through the ELISA kit (RayBiotech, USA). Absorbance was measured at 450 nm using a microplate reader (Tecan, Crailsheim, Germany) and leptin concentrations were calculated according to the standard curve. EMPs were tested by using circulating using flow cytometry as previously described [36, 37]. To exclude the possibility of the unintended measurement of platelet microparticles, EMPs were defined as microparticles positively labeled by CD31 and negatively labeled by CD42.

### 2.6. EPCs Culture

EPCs were isolated and cultured as previously described [38]. After 4 d culture, nonadherent cells were removed and endothelial cell basal medium-2 (EBM-2) was changed (Lonza, Swiss). Later on, the medium was changed every 3 days. After 3 to 4 weeks' culture, late EPCs were examined by using flow cytometry analysis to identify endothelial markers including CD31, CD34, and CD309 (BD Pharmingen) as previously described [39] and then were incubated in starvation medium with or without 3-methyladenine (3-MA, 5 mM, 2 h).

### 2.7. EPCs Migration In Vitro

EPCs were marked with a “+” on the wall of the six-well plates when there were 80%–90% adherent cells and washed with the PBS to remove the nonadherent cells; images were photographed by a light microscope at that time and after 12 h in the same position for analyzing. Images were obtained using a light microscope at 100x magnification.

### 2.8. EPCs Adhesion In Vitro

EPCs (3 × 10^4^ cells/100 *μ*L) were resuspended at 1mL with EBM- 2 serum-free medium and added to a human fibronectin (FN)-incubated six-well plates. The cells were cultured at 37°C for 5 h, and the nonadherent cells were washed out with PBS. After fixing the cells with 4% paraformaldehyde for 15 min and 0.3% crystal violet for another 15 min, images were obtained using a light microscope at 100x magnification.

### 2.9. EPCs Tube Formation In Vitro

A growth factor-reduced Matrigel (BD Biosciences, USA) was dissolved at 4°C, added to 96-well plates at 80 *μ*l/well, and then incubated at 37°C, 5% CO_2_ for 1 h. 3 × 10^4^ EPCs were resuspended in EBM- 2 serum-free medium at 100 *μ*L and loaded on the top of the Matrigel. After 5 h of incubation, EPC tube formation was assessed by light microscopy and photographed at 40x magnification.

### 2.10. EPCs Starvation in Vitro

For starvation, EPCs were incubated in starvation medium including 20 mM HEPES [pH 7.4], 1% BSA, 140 mM NaCl, 1 mM CaCl_2_ and 1 mM MgCl_2_ for 1.5 h [31].

### 2.11. Autophagy Process Detection

EPCs were transfected with mRFP-GFP-LC3 adenovirus obtained from Hanbio (Biotech, Shanghai, China). Confocal fluorescence microscopy (ZEISS, German) was used to evaluate autophagy flux by counting the cells with GFP-LC3 (green) puncta, RFP-LC3 (red) puncta, and GFP+/mRFP+ -LC3 (yellow) puncta.

### 2.12. Western Blot Analysis

Total EPC protein was extracted and quantified by RIPA Lysis Buffer (Beyotime Biotechnology, China) containing protease inhibitors (Roche) and BCA assay kit (I Thermo, USA) separately. Protein extracts were separated by SDS-PAGE, transferred to PVDF membranes (Roche, Indianapolis, IN, USA). The following antibodies were used: anti-Beclin1 antibody (1 : 1000; ImmunoWay, USA), anti-Atg5 antibody (1 : 1000; ImmunoWay, USA), rabbit anti-LC3 antibody (1 : 1000; Cell Signaling Technology, USA), and anti-ACTB antibody (1 : 1000; Cell Signaling Technology, USA). Proteins were visualized with HRP-conjugated anti-rabbit IgG or anti-mouse IgG (1 : 3000; Cell Signaling Technology, USA), followed by the use of the ECL chemiluminescence system (Thermo). And the level of protein was analyzed by using Image J.

### 2.13. Statistical Analysis

All results were expressed as mean ± standard deviation (SD). The normal distribution of continuous variables was determined via the Shapiro–Wilk test. Two groups of normal distribution data were performed using the Students *t*-test and Wilcoxon test for nonnormal distribution data. Statistical significance of multiple groups was assessed by one-way ANOVA for normal distribution data and followed by Bonferroni test. They were two-tailed. *P* < 0.05 represented statistically significant. All statistical analyses were performed in Statistical Package for Social Sciences version 23.0 software (SPSS Inc., Chicago, IL, USA).

## 3. Results

### 3.1. Baseline Characteristics and Changes after FT

A total of 13 subjects received FT intervention in this pilot study (see Table 1). Results showed that there were significant reductions in body weight (81.76 ± 12.04 vs. 77.51 ± 12.06 kg; *P* < 0.01), BMI (29.93 ± 2.82 vs. 28.47 ± 2.83 kg/m^2^; *P* < 0.01) after FT; and bodyweight decreased approximately 4.0 kg in average after 7 days, besides fasting plasma glucose (FPG, 5.25 ± 2.24 vs. 3.90 ± 0.52 mmol/L; *P*=0.04) and systolic blood pressure (SBP, 135.8 ± 17.39 vs. 125.3 ± 15.01 mmHg; *P* < 0.01). As for lipid metabolism, triglycerides (TG, *P*=0.03) decreased, whereas total cholesterol (TC, *P*=0.01), low density lipoprotein cholesterol (LDL-C, *P*=0.01) increased, but TC and LDL-C would return to baseline or even lower after getting back to normal diets (unpublished data); it may be due to the steatosis and energy supply during fasting time. The exact mechanism deserves further investigation. All the participants went through FT without hypoglycemia or other severe adverse events. No one showed liver or renal impairment.

### 3.2. Endothelial Function and Arterial Stiffness Response to FT

**Figure 2:**
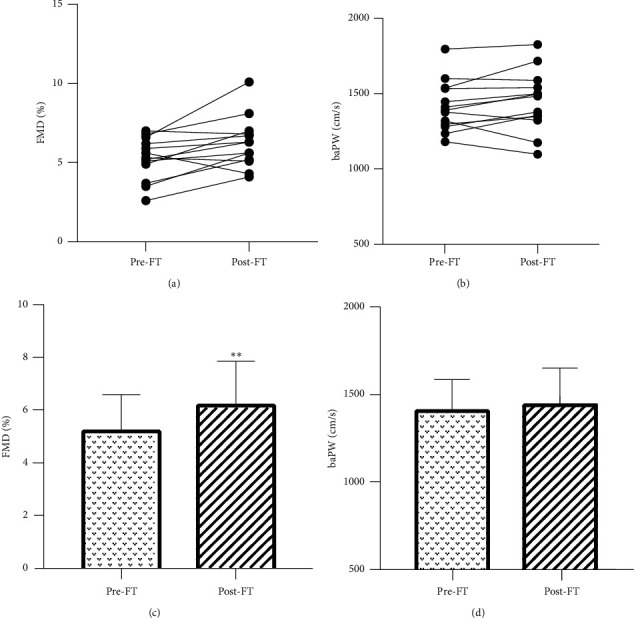
Effects of FT on FMD and baPWV. (a) Individual changes in FMD. (b) Individual changes in baPWV. (c) The mean value of FMD. (d) The mean value of baPWV. ^*∗*^*P* < 0.05, ^*∗∗*^*P* < 0.01. FMD: flow-mediated dilation; baPWV: brachial-ankle pulse wave velocity.

FMD and baPWV are the most reproducible measures to indicate endothelial function and arterial stiffness which are widely used in clinical research. It showed FT ameliorated endothelial function after 7-day intervention. Overall, FMD significantly increased (5.26 ± 1.34 vs. 6.25 ± 1.60%, *P*=0.01; see Figures 2(a) and 2(c)). baPWV had no statistically significant change (1416 ± 168.5 vs. 1449 ± 201.6 cm/s, *P*=0.22; see Figures 2(b) and 2(d)).

### 3.3. Circulating TMAO, Leptin, and EMPs Response to FT

**Figure 3:**
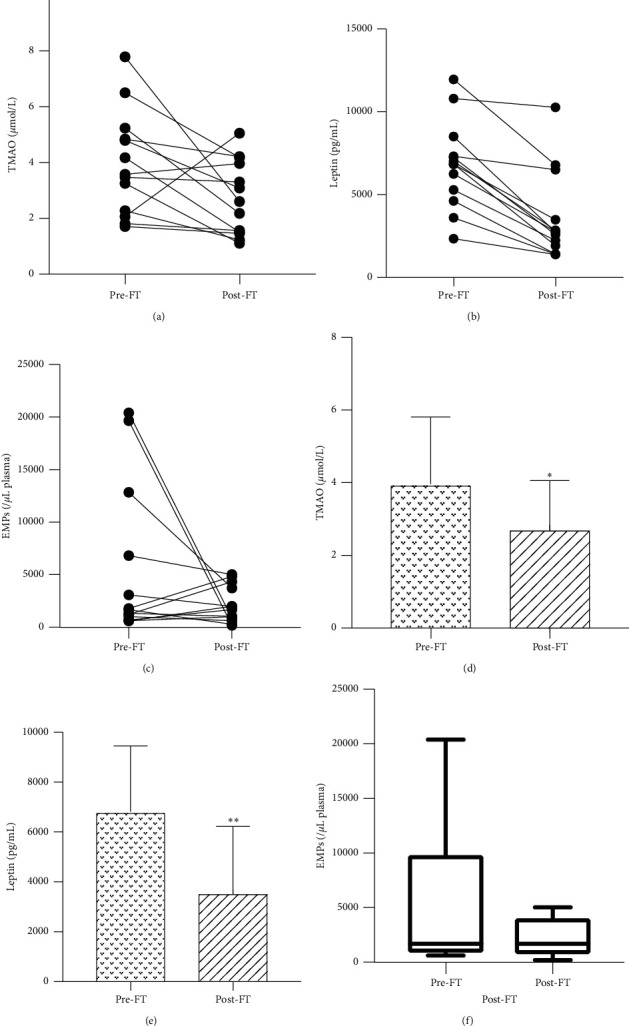
Effects of FT on circulating TMAO, leptin, and EMPs concentrations. (a) Individual changes in TMAO. (b) Individual changes in leptin. (c) Individual changes in EMPs. (d) The mean level of TMAO. (e) The mean level of leptin. (f) Minimum to maximum EMPs (nonnormal distribution data). ^*∗*^*P* < 0.05, ^*∗∗*^*P* < 0.01. TMAO: trimethylamine N-oxide; EMPs: endothelial microparticles.

TMAO, leptin, and EMPs all varied among different subjects at baseline (minimum TMAO: 1.71 *μ*mol/L, maximum TMAO: 7.79 *μ*mol/L; minimum leptin: 2332.45 pg/mL, maximum leptin: 11962.49 pg/mL; minimum EMPs: 609.20/*μ*L plasma, maximum EMPs: 20415.90/*μ*L plasma; see Figures 3(a)–3(c)). Mean level of TMAO was significantly lower after FT (3.96 ± 1.85 vs. 2.73 ± 1.33 *μ*mol/L, *P*=0.04; see Figure 3(d)), as well as leptin (6814 ± 2639 vs. 3563 ± 2668 pg/mL, *P* < 0.01; see Figure 3(e)). There was a tendency to the reduction of EMPs; no statistical significance was found (1675 (862.4, 9824) vs. 1697 (747.7, 4035)/*μ*L plasma, *P*=0.38; see Figure 3(f)).

### 3.4. Culture and Identification of EPCs

**Figure 4:**
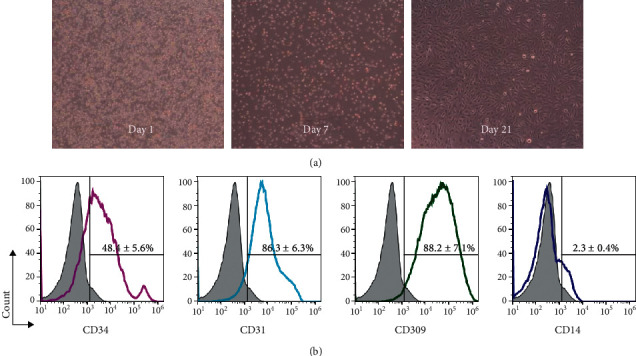
Identification of EPCs. (a) The morphology of late EPCs (100x magnification). (b) The late EPCs markers labeled by flow cytometry.

To investigate the effects of starvation on EPCs function, we isolated and cultured late EPCs from participants' peripheral blood. After about 3 to 4 weeks of culture, the morphology of late EPCs closely approximate mature endothelial cells (see Figure 4(a)). The markers of late EPCs were detected by flow cytometry (see Figure 4(b)), in which CD34 (48.4 ± 5.6%), CD31 (86.3 ± 6.3%), CD309 (88.2 ± 7.1%) increased, and monocyte marker CD14 were significantly reduced (2.3 ± 0.4%); it was similar with the previous study.

### 3.5. The Effects of Starvation on EPCs Function In Vitro

**Figure 5:**
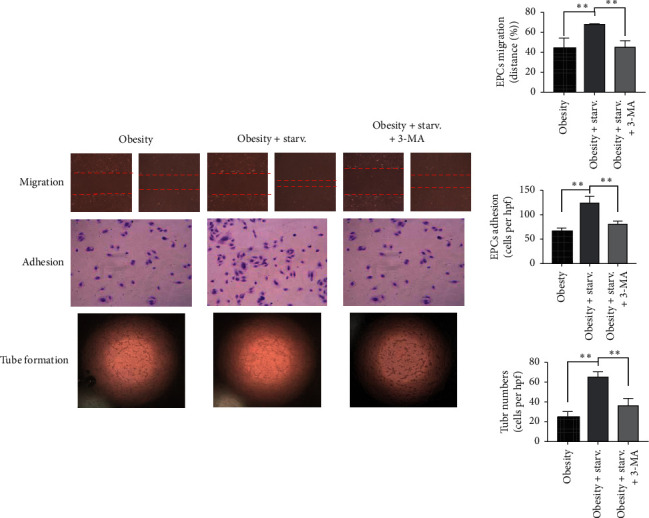
The function of EPCs in different conditions. EPCs with starvation showed an improvement in migration (100x magnification), adhesion (100x magnification), and tube formation (40x magnification) compared with nonstarvation group, and 3-MA blocked the beneficial effects of starvation, the effects of starvation on autophagy of EPCs in vitro.

EPCs were incubated in starvation medium for 1.5 h. The migration (*P* < 0.01), adhesion (*P* < 0.01), and tube formation (*P* < 0.01) of EPCs were all improved compared with the nonstarvation group (see Figure 5). These data showed starvation ameliorated EPCs function.

**Figure 6:**
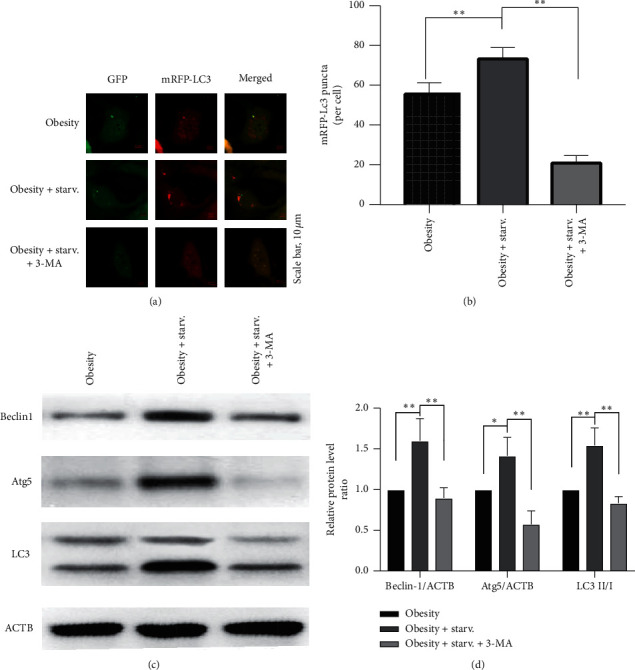
Autophagy level of EPCs. (a) The autophagy fluorescence intensity under a confocal microscope. (b) The mean level of fluorescence intensity. (c) The protein bands of Beclin1, Atg5, and LC3. (d) The mean level of Beclin1/ACTB, Atg5/ACTB, and LC3 II/I ratio.

EPCs were transfected with mRFP-GFP-LC3 adenoviruses to detect the autophagy process (see Figure 6). The fluorescence intensity of LC3 was enhanced in EPCs with starvation compared with nonstarvation group (*P* < 0.05), paralleled with the increase of protein levels of Beclin1 (*P* < 0.01), Atg5 (*P* < 0.01), and LC3 (*P* < 0.01). In addition, 3-MA, the autophagy inhibitor, blocks the effects of starvation in improving EPCs function (see Figure 5).

## 4. Discussion

This pilot study explored the effects of FT on arterial function, vascular injury-related biomarkers, and possible mechanisms in overweight and obese individuals. Apparently, FT safely and effectively improved FMD and reduced body weight among overweight and obese individuals, whereas baPWV showed no marked change. Meanwhile, FT reduced vascular injury-related circulating biomarkers such as TMAO and leptin, and EMPs exhibited a descending trend but with no statistical significance. In vitro, starvation improves EPCs ability of migration, adhesion, and angiogenesis via activating autophagy, and inhibition of autophagy eliminated the improvement of EPCs function. To the best of our knowledge, these data demonstrated for the first time that FT reduces vascular risk through improving endothelial function and reducing vascular injury-related biomarkers in overweight and obese individuals via upregulating EPCs function and autophagy level.

For overweight and obesity, calorie-restricted weight management is recommended as the cornerstone of staying healthy [40]. Fasting is a procedure that a person voluntarily goes without food for a certain length of time, living mainly on their fat reserves [18]. L-carnitine is a conditionally essential nutrient necessary for transporting long-chain fatty acids into mitochondria in order to produce energy, especially during fasting [41]. Our previous studies have proved that intravenous L-carnitine supplementation during the fasting period could ameliorate adverse reactions such as hunger and fatigue and help patients insist on the intervention [19, 20]. In recent years, FT has been identified as a mature and effective way of very low calorie diet on weight loss and reduction of cardiovascular risk factors [19].

Animal studies support the concept that calorie restriction has a beneficial impact on the vasculature. Life-long calorie restriction in mice was indicated to prevent endothelial dysfunction and arterial stiffness [42]. However, the impact of periodic fasting therapy on endothelial function in humans remains largely unknown. There were only limited data about the effect of intermittent fasting on endothelial function. An 8-week alternate day fasting study suggested a beneficial effect on insulin resistance, but not endothelial function [43]. Another 4 week 5 : 2 intermittent energy restriction diet strategy (two consecutive very low energy intake days) displayed no improvement on FMD [44]. The present data reported in our study showed that FMD significantly increased by about 1.26% after 7-day fasting intervention. A notable meta-analysis shows a 13% decrease in the future risk of ASCVD for every 1% increase in FMD [45]. Hence, FT is able to significantly improve vascular endothelial function in overweight and obese individuals and might reduce the risk of ASCVD.

Although some evidence demonstrates that modest weight loss (8% of initial body weight) achieved with diet and lifestyle interventions seems to improve PWV [46], our study failed to see any profitable change in baPWV. As baPWV is related to many factors, especially vascular structure [47], the beneficial effect on vascular stiffness may need long-term lifestyle management.

In addition to improvements in endothelium-dependent vasodilation, FT also led to a decline in systemic levels of vascular injury-related biomarkers including TMAO and leptin. TMAO has been established as a predictor of cardiovascular events which is involved in the progress of atherogenesis, endothelial dysfunction, and metabolic disorders and significantly elevated in high BMI individuals [48]. And to our knowledge, this is the first time to find that FT significantly reduced TMAO. High level of circulating leptin enhanced the risk of ASCVD in overweight or obesity [11, 13] through promoting endothelial dysfunction and the expression of profibrotic markers in the heart [13]. Data in our present study also found that FT reduced serum leptin levels. Endothelial cells produce EMPs, a surrogate marker of endothelial injury, in response to cellular dysfunction and predict cardiovascular events [14, 49]. Our study showed a descending trend on the level of EMPs whereas with no statistical significance.

Large amounts of studies demonstrate that EPCs are pivotal to the maintenance of vascular homeostasis [22] and deficiency in function and number of EPCs contribute to endothelial dysfunction in obesity [50, 51]. There was preliminary evidence indicates that restrict calorie restriction increased endothelial function in vivo; a 2-day fasting intervention improves endothelial progenitor cell-mediated ischemic angiogenesis in mice [52]. In our study, we found starvation of obese EPCs in vitro improved the abilities of migration, adhesion, and angiogenesis.

Autophagy is a highly conserved and complex process to preserve cellular homeostasis involved in many functional proteins. Beclin-1 is a key factor in the formation of autophagy, and Atg5 and LC3 participate in the extension of the autophagic membrane to identify and wrap damaged proteins and organelles, eventually form mature autophagosomes [53]. Studies showed that autophagy defect is associated with endothelial dysfunction of patients with various metabolic disorders including obesity [26, 28]. Fasting or calorie restriction is a classical and acknowledged method to activate autophagy [29–31]. Our previous studies found that FT improved insulin resistance in obese rats by activating Beclin1/LC3-mediated autophagy. The present data in this study showed that starvation enhanced the fluorescence intensity of LC3 and increased the level of autophagy-related proteins such as Beclin1, Atg5, and LC3 II/I. It is worth mentioning that 3-MA, the autophagy inhibitor, blocked the beneficial effects of starvation on obese EPCs function.

The study still has several limitations. First, this work represents a small, interventional pilot study designed to determine whether FT has favorable effects on arterial function and the levels of vascular injury-related biomarkers in overweight and obese individuals. Surely, a larger, placebo-controlled randomized trial with a long time follow-up is necessary to validate our current findings. Second, whether FT would reduce the morbidity and mortality of ASCVD remains to be investigated in clinical practice. At last, the deeper mechanism of autophagy behind the favorable effects of FT on vascular homeostasis remains unclear and needs further exploration.

## 5. Conclusions

Our present work demonstrated for the first time that FT improves endothelium-dependent vasodilation and reduces vascular injury-related biomarkers in overweight and obese individuals through improving EPCs function via activating autophagy of EPCs. Overall, these findings showed that FT may be an effective therapeutic method to protect vascular health in overweight and obese individuals and retard the incidence of ASCVD in clinical practice.

## Figures and Tables

**Table 1:** Subject characteristics before and after FT interventions.

Subject characteristics	Pre-FT	Post-FT	*P* value
Age, y	43.54 ± 10.60	—	—
Male, *n*	5	—	—
Female, *n*	8	—	—
Overweight, *n*	8	—	—
Obesity, *n*	5	—	—
Body weight, kg	81.76 ± 12.04	77.51 ± 12.06	<0.01^*∗∗*^
BMI, kg/m^2^	29.93 ± 2.82	28.47 ± 2.83	<0.01^*∗∗*^
ΔWeight, kg	—	4.25 ± 1.01	—
Percentage of weight loss, %	—	5.30 ± 1.40	—
Heart rate, per min	68.76 ± 9.68	70.92 ± 9.34	0.40
SBP, mmHg	135.80 ± 17.39	125.30 ± 15.01	<0.01^*∗∗*^
DBP, mmHg	80.54 ± 12.86	80.77 ± 8.97	0.91
FPG, mmol/L	5.25 ± 2.24	3.90 ± 0.52	0.04^*∗*^
TC, mmol/L	5.25 ± 0.94	5.70 ± 1.25	0.01^*∗∗*^
LDL-C, mmol/L	3.17 ± 0.96	3.77 ± 0.91	0.01^*∗∗*^
HDL-C, mmol/L	1.31 ± 0.52	1.11 ± 0.24	0.27
TG, mmol/L	1.71 ± 0.53	1.24 ± 0.38	0.03^*∗*^

BMI: body mass index; SBP: systolic blood pressure; DBP: diastolic blood pressure; FPG: fasting plasma glucose; TC: total cholesterol; LDL-C: low-density lipoprotein cholesterol; HDL-C: high-density lipoprotein cholesterol; TG: triglycerides. Data are shown as n or mean ± SD. ^*∗*^*P* < 0.05, ^*∗∗*^*P* < 0.01.

## Data Availability

The data used to support the findings of this study are available from the corresponding author upon request.
